# Right Occipital Cortex Activation Correlates with Superior Odor Processing Performance in the Early Blind

**DOI:** 10.1371/journal.pone.0071907

**Published:** 2013-08-14

**Authors:** Laurent Renier, Isabel Cuevas, Cécile B. Grandin, Laurence Dricot, Paula Plaza, Elodie Lerens, Philippe Rombaux, Anne G. De Volder

**Affiliations:** 1 Institute of Neuroscience (IoNS), Université Catholique de Louvain, Brussels, Belgium; 2 Escuela de Kinesiología, Pontificia Universidad Católica de Valparaíso, Valparaíso, Chile; 3 Department of Radiology, Cliniques Universitaires Saint-Luc, Brussels, Belgium; 4 Department of Otorhinolaryngology, Cliniques Universitaires Saint-Luc, Brussels, Belgium; University of Montreal, Canada

## Abstract

Using functional magnetic resonance imaging (fMRI) in ten early blind humans, we found robust occipital activation during two odor-processing tasks (discrimination or categorization of fruit and flower odors), as well as during control auditory-verbal conditions (discrimination or categorization of fruit and flower names). We also found evidence for reorganization and specialization of the ventral part of the occipital cortex, with dissociation according to stimulus modality: the right fusiform gyrus was most activated during olfactory conditions while part of the left ventral lateral occipital complex showed a preference for auditory-verbal processing. Only little occipital activation was found in sighted subjects, but the same right-olfactory/left-auditory-verbal hemispheric lateralization was found overall in their brain. This difference between the groups was mirrored by superior performance of the blind in various odor-processing tasks. Moreover, the level of right fusiform gyrus activation during the olfactory conditions was highly correlated with individual scores in a variety of odor recognition tests, indicating that the additional occipital activation may play a functional role in odor processing.

## Introduction

When early deprived of its natural inputs a sensory cortex starts receiving and processing inputs in the remaining modalities [1]–[7]. In humans affected by early blindness (EB), several regions within the occipital cortex (OC) are recruited during the processing of auditory [8]–[13] and tactile stimuli [14]–[23] as well as during various cognitive tasks such as mental imagery [24]–[25], working memory [26]–[27] verbal processing and memory [28]–[32]. More recently, a stronger recruitment of the occipital cortex was also found during odor detection in blind compared to sighted participants [33], indicating further that the so-called “visual” cortex in the blind acquires non-visual functions. While these functional studies of brain activity elucidate some heightened auditory or tactile skills in blind individuals (e.g., enhanced spatial localization) [9]–[10], [13], [34]–[38], there were relatively few attempts to compare, in the same blind subjects, the brain activity elicited by stimuli processed in different sensory modalities [20], [23], [39]. For that reason, it is still unclear to what extent distinct (non-visual) sensory modalities are segregated in the reorganized occipital cortex of blind subjects. In addition, little is known about the cerebral network that supports performance in higher-level odor-processing in early blind individuals (e.g., odor discrimination, categorization or identification) since the few available studies [33, 40) were acquired during passive olfactory stimulation or during odor detection. We previously demonstrated that EB individuals were better than sighted subjects at discriminating, categorizing and identifying odors [41] see also [42]–[43], raising the question of the neural substrate that underlies this improved performance. In order to address the functional plasticity associated with olfactory expertise in the blind, we tested whether the occipital cortex of EB subjects was recruited during higher-level odor-processing tasks and if so, whether olfactory and auditory-verbal processing was dissociated in this cortex. We also tested to what extent the degree of occipital cortex recruitment, if present, would be predictive of individual differences in the behavioral performance of odor-processing tasks.

## Materials and Methods

### Ethics Statement

All participants provided their written informed consent prior to the study according to the Declaration of Helsinki (BMJ 1991; 302: 1194). The experimental protocol of the study was approved by the Biomedical Ethics Committee of the school of Medicine of the Université catholique de Louvain.

### Subjects

The study was carried out in ten early blind subjects (EB, range 23–57 years, mean ± SD: 39.5±11.07) and ten sighted control participants (SC) who were blindfolded during the experiments and matched for age, sex, handedness and educational level (range 22–57 years, mean ± SD: 39.3±11.08, *p*>0.05). All subjects were right-handed males (see Table 1 for additional details regarding the subjects). They were healthy, without recorded history of neurological or psychiatric problems, and without olfactory disorders. Clinical hyposmia was excluded in all EB and 8 of their matched SC subjects in a previous behavioural study [41], [44] whereas all subjects rated their olfactory function as “normal” in self-reported ratings (i.e. being asked to evaluate their olfactory function as either “decreased”, “normal” or “over average). EB subjects were all affected by total blindness (without residual light perception) as a result of bilateral ocular or optic nerve lesions at birth or within the first 2 years of life, well before the completion of visual development [45]. None of the subjects reported any form of visual remembering. In addition, they were all autonomous and well integrated socially.

**Table 1:** Profile of the blind subjects.

*Subjects*	*Age (years)*	*Educational level*	*Onset of blindness*	*Diagnosis*	Performance (**)
EB1	23	Some college	Birth	Persistent hyperplastic primaryvitreous involving both eyes	NA
EB2	28	High school	Birth	Genetic (*)	74 (°)
EB3	31	College degree	Birth	Leber congenital amaurosis	66
EB4	42	High school	Birth	Retinopathy of prematurity	68
EB5	57	College degree	<18 months	Bilateral retinoblastoma	73 (°)
EB6	31	College degree	Birth	Anterior chamber cleavagesyndrome (Peters syndrome)	73
EB7	43	High school	Birth	Unknown, postmature birth (*)	66
EB8	40	College degree	Birth	Premature birth	76
EB9	52	College degree	Birth	Severe retinal dystrophy (*)	72
EB10	48	High school	<24 months	Bilateral retinoblastoma	68

Note: EB: early blind; all subjects were male and right handed; (*): no additional details available.

(**) Behavioral performance in a variety of higher-level odor processing tasks (global percentage of correct answers) before fMRI (see text and Table S1 for details and scores of each task). (°) Behavioral performance in age-matched control was not available for this subject. As subjects EB5 and EB10 had very poor vision from birth and underwent a bilateral eye enucleation by the age of 18–24 months, they were considered early blind. They did not remember any visual experience.

#### Behavioral measurements

We assessed the subject's ability to discriminate, categorize and identify a variety of odour samples before the fMRI study. The material used to test the individual ability in odour-recognition, previously described in detail [41], consisted of 30 commercially available fragrances of flower, fruit, plant or domestic element (http://www.sentosphere.fr). Subjects were examined in sitting position in a well-ventilated dimly lit experimental room. For bi-rhinal stimulations, odorants were presented to the subject one by one with the bottle placed at 2 cm in front of nostrils during 3 s. with a 5 s. time interval between individual odours. A first discrimination task required the subject to smell each stimulus of pairs of stimuli, which had been constituted pseudo-randomly, and to determine whether the second odour was the same (half of the pairs) or different with respect to the first one. A second task of a free-identification was then used, in which subjects had to smell each odorant and name it. No feed-back was provided to the subject about the accuracy of his response before the end of a last task of categorization in which the subject was required to categorize each stimulus in one out of four (provided) semantic categories: fruit, flower, plant or other. The quotation was made on a 0/1 basis, with the total number of correct responses providing the score for each task. These scores were further averaged to provide the global odour-recognition performance (see Table S1 for a list of individual scores).

### Equipment and Stimuli

#### Olfactory stimulation

We used four chemical odorants (Sigma Aldrich®, Germany) as fruit and flower odors: lemon (3,7-Dimethyl-2,6-octadienal dimethyl acetal), banana (Isoamyl acetate), lavender (1-Octen-3-yl acetate), and rose (2-Phenylethyl alcohol). A computer-controlled, MRI-compatible odor delivery system [46] was used as olfactory stimulator that allowed birhinal and timed delivery of each odor in synchrony with MRI sequences and participant’s inspiration phase.

#### Auditory stimulation

We used eight pre-recorded auditory-verbal stimuli (fruit and flower names): kiwi, cherry, tulip, hyacinth, peach, grape, begonia and orchid. An MRI compatible, high-definition piezoelectric sound delivery system was used as audio-system (the so-called SDS device, the fMRI.pl group, http://www.fmri.pl).

### Tasks and Procedure

We used a block design paradigm with four experimental conditions (duration: 21 seconds) alternating with resting state periods (12 seconds) in eight runs of 408 seconds each. The fMRI acquisitions took place during two separate sessions of 4 runs each during the same week. Each condition was briefly announced via headphones during the preceding resting period. The order of conditions was pseudo-randomized and counterbalanced across subjects. Subjects provided their responses using two response button pads, one held in each hand. SC subjects were blindfolded during the experiments.

We used two different tasks in each modality: a categorization task that required an access to semantic information and a discrimination task that supposed a lower-level processing.

#### Odor categorization

Four olfactory stimulations were presented successively in one active block. At each trial, subjects had to categorize the stimulus as a flower or as a fruit. *Odor discrimination*: Four olfactory stimulations were presented successively and subjects had to determine whether each stimulus was the same or different from the preceding one. *Word categorization:* Eight auditory-verbal stimuli (fruit or flower names) were presented via headphones in one active block and subjects had to categorize each stimulus as a flower or as a fruit. *Word discrimination:* Eight stimuli were presented and subjects had to determine whether each word was the same or different from the preceding one. Subjects had to provide their response directly after each stimulus presentation (and before the presentation of the following stimulus). The stimulus sequences were pseudo-randomized.

#### Breathing control

**Figure 1:**
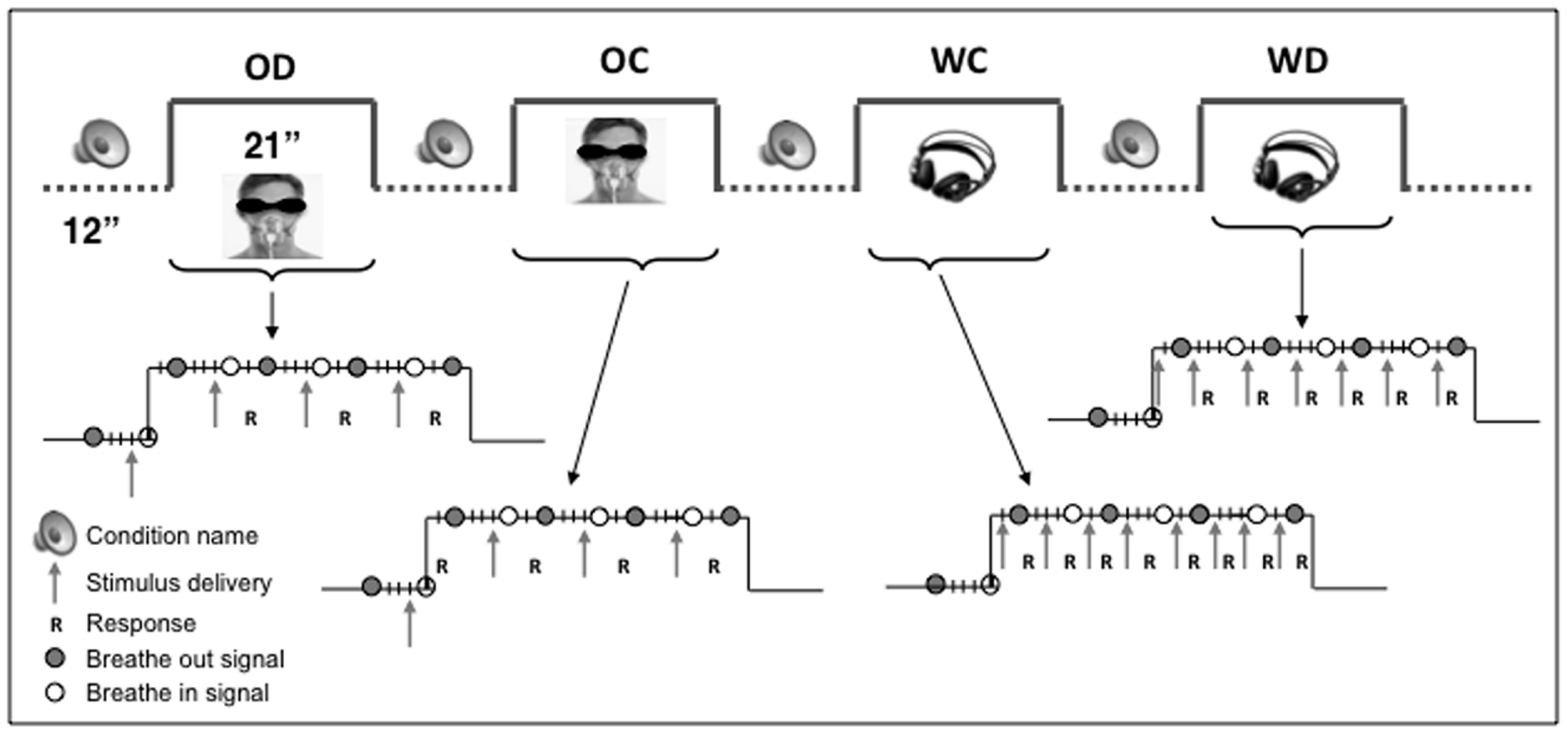
Experimental design. The experimental paradigm consisted in a block design with four experimental conditions (21 seconds) alternating with resting state periods (12 seconds): odor discrimination (OD), odor categorization (OC), word discrimination (WD) and word categorization (WC). Each condition was briefly announced during the preceding rest period. The diagrams illustrate the sequence of events occurring in each active epoch of the paradigm. The circles indicate when auditory signals were provided to inform the subject when to breathe in (empty white circle) and when to breathe out (plain gray circle). The arrows indicate when stimuli were delivered. R: subject response expected.

To synchronize the olfactory stimulation with the inspiration phase, auditory signals were delivered to the subjects to control the breathing (see Figure 1 and Figure S1 for a display of the experimental design and the set-up). Two pure tones, a high frequency (264 Hz) and a low frequency (132 Hz) of 860 ms each, were used to warn the subject when to start an inspiration (breathing in) and an expiration (breathing out). Each stimulation block began with an inspiration occurring 1 second after the beginning of an olfactory stimulation, which stopped two seconds later. Then, the residual odor was flushed out from the system using clean air while the subject was breathing out. There were four inspiration phases in each epoch. During the auditory-verbal conditions, subjects were controlling their breathing the same way in order to keep the experimental parameters as comparable as possible across conditions.

#### Training

Before the fMRI experiment, each subject underwent two one-hour familiarization sessions to make sure that all subjects could control and adapt their breathing rhythm, perform the task and that the difficulty level of the task was equivalent between groups, i.e. did not constitute a confounding factor. The difficulty level of all fMRI conditions was intentionally set at a low level to insure that all subjects would be able to perform accurately the tasks during the fMRI acquisitions. At the end of the training phase all subjects were able to control their breathing as instructed and to discriminate and categorize the stimuli with a mean accuracy superior to 80%.

### 3D-MRI and fMRI Acquisition

Structural brain imaging was obtained in all subjects in the bicommissural (AC-PC) orientation [47] on a 3 Tesla MRI unit (Achieva, Philips Healthcare®, Best, The Netherlands) using a 3D fast T1-weighted gradient echo sequence with an inversion prepulse (Turbo field echo (TFE), TR [repetition time] = 9 ms, TE [echo time] = 4.6 ms, flip angle = 8 degree, 150 slices, 1 mm thickness, in plane resolution = 0.81×0.95 mm). The field of view was 220×197 mm, and the SENSE factor (parallel imaging) was 1.5. We used an 8 channels phased array head coil. Foam pads restrained the head.

Blood oxygen level dependent (BOLD) fMRI data were acquired using a 2D single shot T2*-weighted gradient echo-planar imaging (EPI) sequence (TR = 3000 ms, TE = 27 ms) with 48 axial slices (thickness = 2.4 mm), in the AC-PC orientation. The matrix was 112×112×48 and the field of view was 220 mm^2^. The in-plane resolution was 2.12 mm^2^. The fMRI paradigm consisted in 8 runs of alternating epochs of experimental conditions and rest (21 s per active epoch, 7 brain volume repetitions, alternating with 12 s [4 repetitions] resting periods). Each condition was assessed 3 times in a separate run, in counterbalanced order. The first inspiration phase was synchronized with the acquisition of the first slice of the epoch.

### Data Analysis

Data analysis was performed using the BrainVoyager QX 2.2 software package (Brain Innovation™, Maastricht, The Netherlands) using standard preprocessing procedures. fMRI data preprocessing included head motion correction, slice scan time correction, and high-pass filtering (cutoff frequency: 3 cycles/run) using temporal smoothing in the frequency domain to remove drifts and to improve the signal to noise ratio. No data included in the study exceeded motion of 2 mm in any given axis or had spike-like motion of more than 1 mm in any direction during a given fMRI session. Functional and anatomical data sets for each subject were coregistered and the resulting matching brain images were fit to standardized Talairach space [47]. Single-subject functional data were spatially smoothed (4 mm FWHM) in order to reduce intersubject anatomical variability and analyzed using one multiple regression model (General Linear Model, GLM [48]) consisting of predictors, which corresponded to the four experimental conditions, and in which the beta weights quantify the potential contribution of the predictors in explaining each voxel time course. The predictor time courses of the regression model were computed on the basis of a linear model of the relation between neural activity and hemodynamic response, assuming a rectangular neural response convolved with standard hemodynamic response function (HRF) during phases of active conditions [48]–[50].

A random-effects (RFX) group analysis [51] was then performed at the whole-brain level, using a mask for the cortex (gray matter) using one-sample and two-sample t tests with a threshold of p<0.001, uncorrected, in combination with a cluster size threshold adjustment to achieve a corrected p<0.05. This was done based on the [52] Monte Carlo simulation approach, extended to 3D data sets using the threshold size plug-in Brain Voyager QX [53]. In order to find the areas activated in each modality (olfactory and auditory-verbal processing), two basic contrasts of interests (compared to rest) were explored, both within and between groups: {olfactory conditions} (contrast weight : [1 1 0 0]) (areas involved in two aspects of active odor processing, i.e. odor discrimination and categorization), {auditory-verbal conditions} [0 0 1 1 ] (areas involved in the same tasks using auditory-verbal stimuli) and their interactions were tested at the whole brain level. In addition, to measure the link between performance and brain activity, a covariance analysis was performed on {olfactory conditions} brain activation maps with the individual odor-recognition score (averaged score for discrimination, free identification and categorization, see Table S1) used as the covariate. In order to control for group size, only 16 subjects (8 SC where this score was available and their matched EB subjects) were included in the covariance analysis. In all the areas with {olfactory conditions} where brain activity was found to be higher in EB group compared to SC group, correlation analyses were also performed between the beta weights of {olfactory conditions} and the performance values (odor-recognition scores), in order to identify the key area(s) explaining performance disparity between EB and SC subjects.

## Results

### Behavioral Results

The behavioural study showed that EB participants were significantly better to discriminate, categorize and identify odours than SC subjects (ANOVA’s: all p’s <0.05 in the group comparisons: p<0.005, <0.001, <0.01 for discrimination, identification and categorization respectively) and, as a consequence, had higher global odour-recognition scores (p<0.001). The mean scores for correct responses in EB and SC groups were 28.4±0.9 and 25.4±2.2 for odor discrimination, 13.0±1.9 and 6.4±1.5 for free identification and 22.4±1.9 and 19.0±2.8 for categorization. Individual scores for each task are shown in Table S1. The recorded in-scan responses confirmed that all subjects could perform adequately and easily the tasks (above 80% in all the subjects in all four conditions).

### Functional Imaging Results

#### Olfactory and auditory-verbal activation patterns

In the entire group (both EB and SC plotted together), the contrast {Olfactory conditions *vs*. Rest} showed the expected bilateral activation in the olfactory cortex: in the entorhinal cortex (Brodmann area [BA] 34), the amygdala, the orbito-frontal cortex (OFC, BA10 and BA11) and the insula/OFC (BA13-47) (see Figure S2 for a display of brain activation related to olfactory processing). The contrast {Auditory conditions *vs*. Rest} showed bilateral activation in the primary and secondary auditory cortices within the superior and transverse temporal gyri (BA 41, 42, 22) (see Figure S3 for a display of brain activation related to auditory-verbal processing). No group difference was observed in any of these olfactory and auditory regions (all *p*’s>0.05 uncorrected), though there was a trend toward stronger activation in SC subjects in the left auditory cortex only (*p* = 0.06 uncorrected) (Figures S2 and S3).

During both olfactory and auditory conditions the occipital cortex was largely activated in EB subjects and to a much lesser extent in SC subjects (see Figure S4). These activation foci were mostly located within the ventral part of the occipital cortex and included the fusiform gyrus, the lingual gyrus and inferior and middle occipital gyri. In addition, we observed an opposite hemispheric lateralization for the olfactory and auditory processing. In the two groups, the left hemisphere was dominant for the auditory-verbal processing while the right hemisphere appeared more activated than the left one during the olfactory processing.

#### Group comparison and modality-specific activation

**Figure 2:**
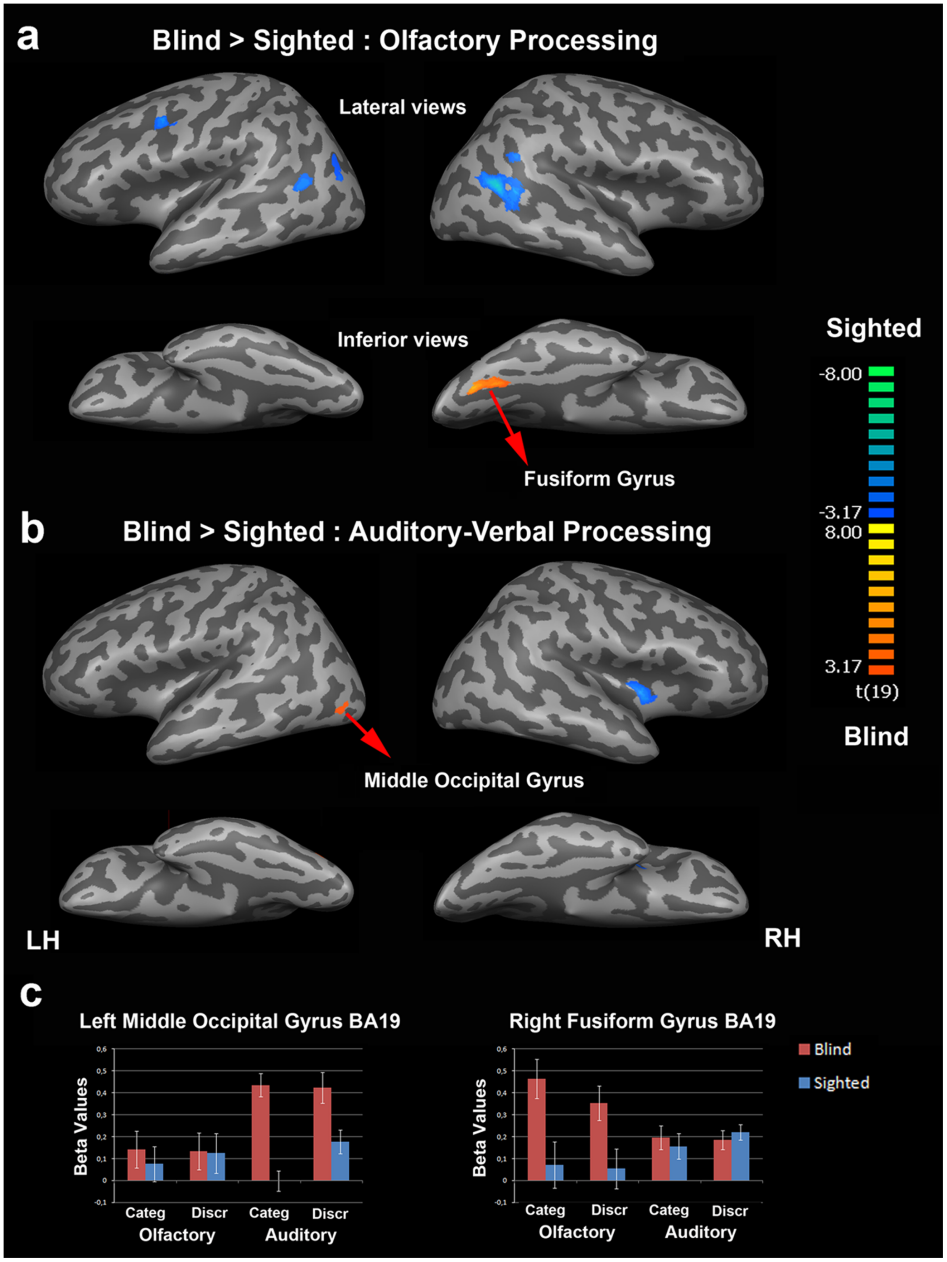
Between-group comparisons for olfactory and auditory-verbal processing. (a) Brain regions that were more activated in EB than in SC subjects during olfactory processing are in orange-yellow and those with higher activation in SC than in EB subjects are in blue-green according to the color scale that codes the t-values. (b) Comparison of brain activation patterns in EB and SC subjects during auditory-verbal processing (same color code as in (a)). For display purposes, results were shown using a p<005, uncorrected threshold, with a cluster size threshold of corrected p<0.05. The two main activation foci that were more activated in EB than SC subjects as revealed by the group comparison were the right fusiform gyrus (x = 24, y = −67, z = −13) and the left middle occipital gyrus (x = −46, y = −73, z = −5). Both activation foci were located in the ventral part of the occipital cortex. The activation focus in the left middle occipital gyrus was located in the posterior part of the ventral lateral occipital complex [54]. (c) Brain activity profiles (i.e. beta values as a function of task, modality and group) in the left middle occipital gyrus (left side) and in the right fusiform gyrus (right side) as identified in the group comparisons (see (a) and (b)). In each of these two regions, there was a clear dissociation between the olfactory and auditory-verbal conditions (double dissociation) in EB subjects; the right fusiform gyrus showed a preference for olfactory processing and the left middle occipital gyrus was more activated during the auditory-verbal conditions. Error bars are standard errors of the means (s.e.m.). LH, left hemisphere; RH, right hemisphere.

**Figure 3:**
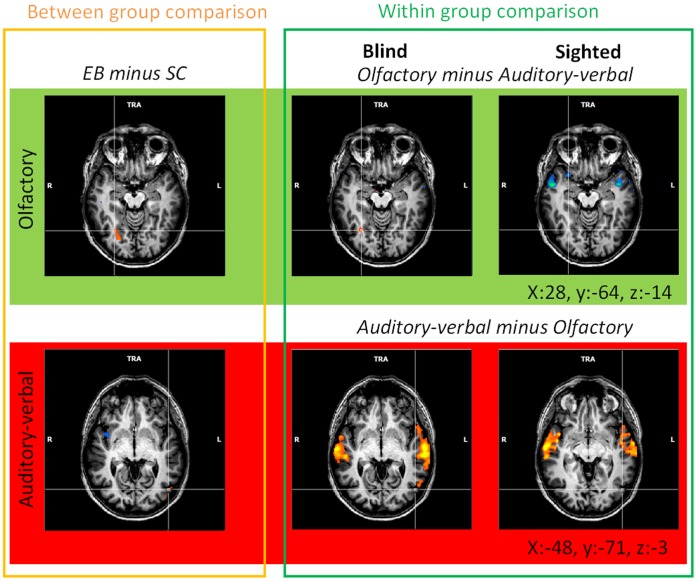
Dissociation between olfactory and auditory-verbal processing in the occipital cortex. Activation maps resulting from a group comparison in each modality (left part of the Figure) and comparisons between the olfactory and auditory-verbal modalities in EB and SC subjects (within group comparison, middle and right parts of the Figure). The activation maps were obtained using random-effects analyses (RFX) with a threshold of *p*<0.001 (uncorrected). Brain activation foci were superimposed on transversal sections of the normalized MRI brain of a representative subject. The crosshairs always intersect on the same voxel in the right fusiform gyrus (FG, x:28, y:−64, z:−14; top of the Figure) and in the left middle occipital gyrus (MOG, x:−48, y:−71, z:−3; bottom of the Figure). Between-group comparisons revealed that the right FG was more activated in EB than in SC subjects during olfactory processing and that the left MOG was more activated in EB than in SC subjects during auditory-verbal processing (see also Figure 2 and Table S2). In EB subjects, within-group comparisons showed that part of the right FG was more activated during olfactory than auditory-verbal processing and that part of the left MOG was more activated during auditory-verbal than olfactory processing whereas such activation foci were not observed in sighted subjects (see Tables S3–4). R: right, L: left.

The group comparisons, performed at the whole brain level in the olfactory and auditory modalities, showed that the occipital cortex was more activated in EB than in SC subjects in both modalities. In the olfactory modality, only one activation focus was found in the right fusiform gyrus (FG, BA 19, x = 24, y = −67, z = −13, 404 voxels) (Figure 2). In the auditory modality, a single activation focus was found in the left middle occipital gyrus (MOG, BA 19, x = −46, y = −73, z = −5, 22 voxels). This activation focus was located in the posterior part of the ventral lateral occipital complex (LOC) [54]. No activation focus was found in the occipital cortex for the reverse contrast (SC *minus* EB) in any of the sensory modalities. This result is in line with the hemispheric lateralization previously found in the main effects related to sensory modality (see Figure S4 for a display of related brain activation in each group). Within-group analyses performed at the whole-brain level in EB subjects (*p*<0.001 uncorrected) revealed activation foci in the occipital cortex of the blind that overlapped those obtained in the group comparisons (see Figure 3, tables S2, S3, S4). The contrasts {olfactory minus auditory} and {auditory minus olfactory} in EB subjects revealed two activation foci: one specific to the olfactory modality in the right fusiform gyrus (x = 29, y = −64, z = −13, 65 voxels) and one specific to the auditory modality in the left MOG (x = −55, y = −61, z = −2, 130 voxels).

#### Covariance and correlation analyses: relationship between olfactory performance and brain activation

**Figure 4:**
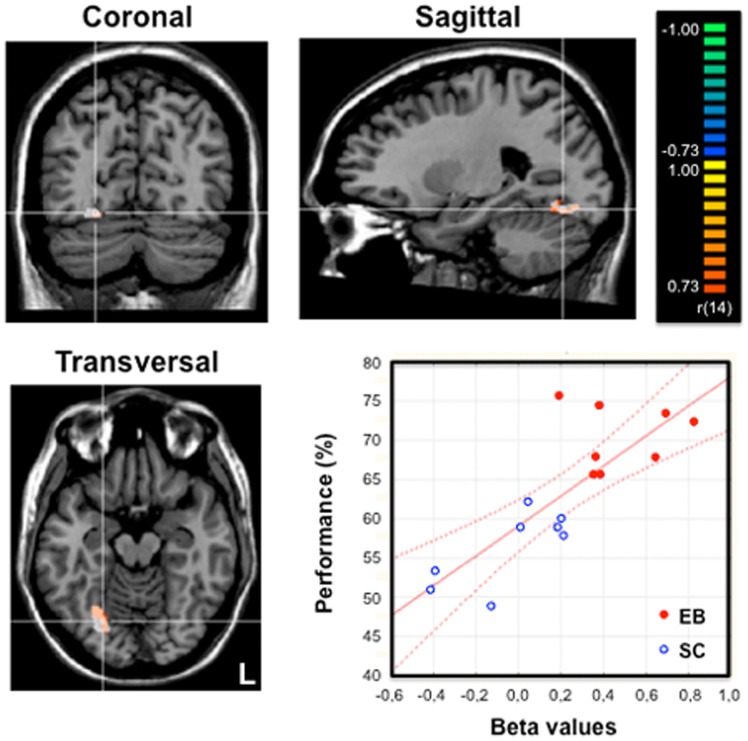
Relationship between olfactory performance and brain activity during odor processing. Activation maps were obtained from an analysis of covariance on olfactory conditions plotted together in 16 (8 EB) subjects using the averaged performance in odor identification, categorization and discrimination as the covariate. Brain regions with a positive covariation were superimposed on the coronal, sagittal and transversal views of a normalized MRI brain of a representative subject. We used a threshold of p<0.001, uncorrected, and a cluster size threshold correction of p<0.05 based on Monte Carlo simulation. An activation focus was found in the right fusiform gyrus (in orange-yellow: 380 voxels, center of gravity: x: 24, y:−64, z: −13) that largely overlapped the brain area previously identified in the group comparison (EB minus SC) for olfactory processing and displayed here in white color as a reference (see also Figures 2 and 3). The crosshairs intersect on a voxel in the right fusiform gyrus (FG, x:24, y:−26, z:−13). The graph at the lower right part of the Figure shows the strong correlation between brain activity (beta weights) in the right fusiform gyrus during odor processing (white region) and the individual performance (averaged score, %) in the whole group of subjects (n = 16): r = 0.80; p<0.001. The red lines indicate the confidence interval (95%). L : left.

The activation map resulting from the analysis of covariance in 16 (8 EB) subjects with the individual global odor-recognition score (composite score : odor discrimination, odor categorization and odor identification) used as the external covariate of interest is displayed in Figure 4, coding the covariation between overall performance in odor recognition and brain activity across the entire brain volume during {Olfactory conditions *vs*. Rest}. Amongst the brain regions activated in the contrast {Olfactory conditions *vs*. Rest} either in EB or in SC subjects, only one brain region showed a positive and significant covariation between the individual performance in odor recognition and brain activity. This brain area was located in the right fusiform gyrus (FG, BA 19, x = 24, y = −64, z = −13, 380 voxels) and largely overlapped the activation focus obtained in the group comparison (EB minus SC) for olfactory processing (see Figure 4).

A complementary correlation analysis performed in these 16 subjects (8 SC and their matched EB subjects) showed a strong relationship between the performance index (odor recognition score) and the beta weights of the fusiform gyrus as identified in the contrast {olfactory conditions in EB minus olfactory conditions in SC} (r = 0,80, p<0.0001, see graph in Figure 4). It is worth noting that the covariance and correlation analyses based on the discrimination, free identification and categorization subscores separately yielded similar results (r = 0,64, p<0.01, r = 0,77, p<0.0001, r = 0,65, p<0.01).

#### Task-related activation patterns

Using a threshold of *p*<0.001 (uncorrected), the task comparisons, {categorization minus discrimination} and {discrimination minus categorization} did not show any activation focus in the occipital cortex of EB subjects in any of the two modalities (grouped together or tested separately).

## Discussion

### Functional Dissociation in the Occipital Cortex of the Early Blind

Here, we found a dissociation between olfactory and auditory-verbal processing in the occipital cortex (OC) of early blind (EB) subjects; the right fusiform gyrus was most activated during the olfactory conditions and part of the left middle occipital gyrus, located in the posterior part of the ventral lateral occipital complex [54]–[55] showed a preference for auditory-verbal processing (Figure 2). In addition, there was a strong correlation between the level of right fusiform gyrus activation during the olfactory conditions and the individual performance in a variety of odor identification tests.

### Methodological Implications

Observing a double dissociation makes it unlikely that the observed differences between olfactory and auditory-verbal conditions were due to a general factor, such as arousal level, attention or difficulty level, which would have provided at most a single dissociation. The same tasks (stimulus discrimination and categorization) were used and the stimuli used in the two modalities belonged to the same semantic categories (fruits and flowers). The olfactory- and auditory-verbal-specific activation foci were spatially well segregated in different hemispheres and brain structures. We can therefore exclude any potential effect of the semantic category or inter-individual variations in the location and extent of the olfactory and auditory-verbal activation foci to explain the observed dissociation. These findings constitute some of the very first evidences that stimuli that are different in their nature recruit different parts of the occipital cortex in the same EB individuals; olfactory and auditory-verbal processing recruits distinct neural networks in this cortex.

### Olfactory and Auditory-verbal Processing in the Occipital Cortex of the Early Blind

In a recent fMRI study, [33] reported occipital cortex (OC) activation in congenitally blind subjects when they paid attention to their olfactory sense to detect odors, but until now, no study had investigated whether distinct parts of the OC of blind subjects were specifically involved in higher order stimulus processing in olfaction as compared to other senses. In EB subjects, a recruitment of the OC during the processing of non visual information (including verbal stimuli) was consistently reported in many studies, although most of them focused either on the auditory or the tactile modality [11], [14], [17], [28], [30], [32], [37]. It has been proposed that the crossmodal recruitment of OC in EB subjects could explain their enhanced perceptual and memory abilities in the auditory and tactile modalities [1], [10], [13], [30], [34]–[35], [38]. Similarly, we show that the OC activation observed in EB subjects during olfactory processing predict their improved performance in various odor identification tasks (Figure 4, Table S1). Only the subjects who had the best scores among the sighted group showed a trend to recruit the right fusiform gyrus, indicating that this brain area could potentially play a role in odor recognition in sighted subjects as well. In the absence of vision, the olfactory sense becomes more crucial to identify persons and places and to evaluate the quality of food before bringing it to the mouth [42]. Because EB people rely more on olfaction, they develop superior abilities to process odors [43]–[44]. The “visual” cortex would provide the neural basis to facilitate the emergence of this type of practice-related behavioral compensation in blind subjects [4].

### Cross-modal Plasticity and Sensory Specialization in the Occipital Cortex

For long the question of the specific/nonspecific nature of the brain activation observed in the OC of EB subjects has been debated. On the one hand, several occipital regions in blind subjects seemed quite indifferently recruited in various experimental conditions (tasks and stimuli) which led some authors to propose that the OC recruitment was nonspecific and served a general purpose [2], [19], [56]. On the other hand, several studies brought evidence for the existence of functional specializations in the OC of blind subjects [23]–[24], [30], [57]–[60]. To reconcile these points of view one could hypothesize that both theories are partly correct. Demonstrating the existence of a modular organization in some parts of the OC in EB does not necessarily exclude that other regions in the OC may support supra-modal or general factors that are involved in various perceptual and cognitive processes. The visual cortex is indeed quite complex and includes numerous specialized modules in sighted subjects [61]–[62] and probably in EB subjects as well.

Here, we showed for the first time a clear dissociation between olfactory and auditory-verbal modalities. This dissociation was found independently to the task performed (e.g. stimulus discrimination and categorization) which suggests that it was mainly driven by the stimulus processing. This finding constitutes new evidence in favour of the existence of functional specialization in the OC of EB subjects and sheds light on how nonvisual modalities are distributed in their reorganized OC. In the absence of visual inputs, nonvisual sensory modalities extend their cerebral networks into the OC to improve perceptual processing and remain, at least partly, segregated in this region. This also indicates that the stimulus nature is a key factor to understand the functional specialization of the OC of the blind [58]. The few studies that compared in the same blind subjects the brain activation elicited by the processing of auditory *versus* tactile stimuli did not find any clear dissociation between these two modalities in the OC [20], [23]. However, the stimuli used in these studies shared close physical properties: both were mechanical waves perceived either via the auditory (sounds) or the tactile (vibrations) modality. By contrast, the odor stimuli (chemical stimuli) used here were clearly different in nature from odor names. Furthermore, unlike the olfactory stimuli, auditory-verbal stimuli involved explicitly a verbal processing though the olfactory conditions may also have involved strategies based on verbalization. Finally, olfaction is the only sense for which there is no thalamic relay before reaching the primary sensory cortex [63].

### Cross-modal Plasticity and Preserved Functional Segregation of the Ventral and Dorsal Streams

Several studies provided evidence in favour of preserved functional specialization within the so-called “visual” streams in EB humans. This was clearly demonstrated for the dorsal stream, where several regions retained their designated functional role in spatial and motion processing regardless to visual experience [22]–[23], [25]–[26], [57], [59]–[60], [64]–[66], and to a lesser extent for the ventral stream [17], [24], [67]. Interestingly, a similar preservation of the functional specialization was recently found in the auditory cortex of early deaf cats [68]–[69]. In the present study, most activation foci observed in the OC of EB subjects in both modalities were found in brain areas usually considered part of the “visual” ventral stream in sighted subjects [62], [70]. Given the nonspatial nature of the two tasks used in the present study, this brought further support to the growing evidence showing that the general functional role of regions in the ventral stream might be retained in blindness, but changes sensory modality [23].

### Cross-modal Plasticity and Hemispheric Specialization in the Occipital Cortex

In the present study, we found opposite brain lateralization for olfactory and auditory-verbal processing in both groups: olfactory processing activated more the right hemisphere while auditory-verbal processing was dominant in the left side of the brain (see Figure S4). This type of lateralization was previously reported in sighted subjects both for olfactory [71]–[73] and verbal processing [74]–[75]. In EB subjects however, this lateralization appeared more pronounced in the OC, while this cortex was little activated in SC subjects. This strengthened lateralization in the OC of the blind leads us to suggest that parts of this cortex become a prominent component of the specialized olfactory and auditory-verbal cerebral networks after early visual deprivation. It is worth noting that a similar left-sided OC lateralization has been recently reported in EB subjects for verbal processing [31]–[32], [76].

### Conclusions

Here we demonstrate that, in the absence of visual input, nonvisual sensory modalities colonize the “visual” cortex and that olfactory and auditory-verbal processing remains segregated in this cortex. Furthermore, the ventral stream seems to develop its designated functional role in processing stimulus identity independently of visual experience. We also demonstrate that the brain activity level in the right fusiform gyrus predicted performance in olfactory identification tasks indicating the specific role of this region in the processing of odors. Additional neuroimaging studies should further investigate in early blind subjects to what extent different sensory modalities are segregated in the occipital cortex and how nonvisual inputs promote development of functional modules within the ventral stream.

## Supporting Information

Figure S1 **Olfactory stimulation equipment.** (A) Image of a blindfolded sighted participant equipped with the odor delivery system in the fMRI room. Auditory signals were delivered via headphones to synchronize odor stimulations and breathing rhythm. (B) Detailed front view of the computer-controlled stimulator device showing the nylon channels, fittings and Teflon tube that deliver the switched air streams to the participant via a removable medical mask, as well as the solenoid valves and oil lubrificators containing the four different odorants in solution [42]. The main part of the device and the computer remained outside the fMRI room, whereas five Nylon channels passed to the fMRI room through a conventional security hole (see [42] for details).
(TIF)

Figure S2 **Brain activation observed during the olfactory processing.** Activation maps resulting from olfactory conditions as compared to the baseline {Odor categorization+Odor discrimination *versus* Rest} in the whole group (both early blind (EB) and sighted control (SC) subjects grouped together: n = 20). To better circumscribe the activation foci in the olfactory regions shown in this Figure, we used a threshold of *q*<0.01 (FDR corrected). Brain activation foci were superimposed on the axial and coronal sections of an individual normalized MRI brain. Significant differences in this contrast (random-effects (RFX) analysis) are coded using a color scale of the t-values. Activation foci were found in the left and right entorhinal cortex/amygdala, which is considered as a part of the primary olfactory cortex [63], [77]–[78] . Additional activation foci were found in the secondary olfactory cortex: the orbito-frontal cortex (OFC, BA11) and the insula/OFC (BA13-47) bilaterally. In the left and right entorhinal cortex, the cluster size were 233 and 17 voxels. The activation foci in the OFC were located in three sub-regions: BA10 in the right hemisphere (x = 37, y = 53, z = 4; 220 voxels), BA11 bilaterally (x = 24, y = 41, z = −10; 333 voxels and x = −28, y = 38, z = −11; 189 voxels) and BA47 bilaterally. The activation foci in BA47 were included in larger clusters that covered most of the insula (x = 37, y = 17, z = 5; 6959 voxels and x = −37, y = 16, z = 7; 4315 voxels). The graphs on the left show the beta values for the olfactory conditions plotted together in four representative olfactory regions (the right entorhinal cortex: x = 17, y = −1, z = −15, 17 voxels, the left entorhinal cortex: x = −19, y = −3, z = −16, 233 voxels, the right OFC (BA11): x = 24, y = 41, z = −10, 248 voxels and the right insula: x = 37, y = 17, z = 5, 2577 voxels) as a function of the group. Error bars are standard errors of the means (s.e.m.). No group difference was observed in any region in the olfactory cortex (all *p* values >0.05, see Results section). Coordinates refer to the referential defined by the atlas of Talairach and Tournoux. R: right; L: left.
(TIF)

Figure S3 **Brain activation observed during auditory-verbal processing.** Activation maps resulting from auditory conditions as compared to the baseline {Word categorization+Word discrimination *versus* Rest} in the whole group (both EB and SC grouped together: n = 20) in random-effects analysis (RFX). To better circumscribe the activation foci in the auditory regions shown in this Figure, we used a threshold of *q*<0.05 corrected for multiple comparisons with a false discovery rate (FDR). Brain activation foci were superimposed on a transversal section of the normalized MRI brain of a representative subject. Activation foci are shown in the left and right primary and secondary auditory cortices (BA41, 42, 22) according to the color scale that codes the t-values. The lines intersect at coordinates (x = −50, y = −25, z = 7) on a voxel in the left transverse temporal gyrus (BA41). The graph on the right shows the beta values for the auditory conditions plotted together in a ROI of 604 voxels centered on the left transverse temporal gyrus (at the line intersection), as a function of the group. Error bars are standard errors of the mean (s.e.m.). No group difference was observed in any region in the auditory cortices though a trend was found in the left side, only (all *p*’s>0.05, see Results section). R: right.
(TIF)

Figure S4 **Brain areas recruited during olfactory and auditory-verbal processing in the group of blind subjects and the control group.** Functional brain activity maps in 10 early blind subjects (EB) and 10 sighted control subjects (SC) during olfactory and auditory-verbal processing were projected onto a 3-D representation (inflated brain) of the right and left hemispheres (RH & LH) of a representative brain of one subject. The activation maps resulting from the contrasts between the olfactory and auditory-verbal conditions compared to the baseline (rest) were obtained using random-effects analyses (RFX) with a threshold of *q*<0.05, corrected for multiple comparisons using false discovery rate (FDR). (a) Brain activation related to olfactory processing in EB (left) and in SC subjects (right). (b) Brain activation related to auditory-verbal processing in EB (left) and in SC subjects (right). During the olfactory and auditory-verbal conditions, the occipital cortex was significantly activated in EB subjects and to a much lesser extent in SC subjects. Most activation foci observed in the OC of EB subjects were located mainly in the ventral stream in both sensory modalities. (c) The graphs show the laterality indexes (left) and the proportion of activated voxels within the occipital cortex relative to the number of activated voxels in the entire brain (right). Laterality indexes were similar to those used to measure the lateralization of functions such as language (e.g. [79]–[80]). The laterality indexes were calculated by dividing “x” by “x+y” (x/x+y), “x” corresponding to the number of voxels located in the right hemisphere as obtained in the contrasts (olfactory *minus* rest) and (auditory-verbal *minus* rest) and “y” corresponding to the number of voxels located in the left hemisphere as obtained in the same contrasts. These laterality indexes were calculated at ten different thresholds (between *p* = 0.05 and *p* = 0.000025) and averaged together. The mean laterality index was then plotted as a function of group and modality in the whole-brain and in the occipital cortex. The averaged laterality index indicates the degree of lateralization in each modality: when comprised between 0 and 0.5 (lower part of the graph) it indicates a left lateralization whereas from 0.5 to 1 (upper part of the graph) it indicates a right lateralization (see arrow). At the whole-brain level, there was an opposite lateralization for the olfactory and the auditory-verbal conditions in both groups; the right hemisphere was dominant for olfaction and the left hemisphere was dominant for auditory-verbal processing. In addition, this lateralization appeared strenghtened in the occipital cortex of EB subjects. The graph on the right shows the proportion (percentage) of activated voxels in the occipital cortex as compared to the number of activated voxels in the entire brain. The proportions of activated voxels in the occipital cortex were obtained using the same contrasts and the same thresholds as those used to calculate the laterality indexes (see above). These percentages of activated voxels were then averaged and plotted as a function of the group and the modality. The proportion of activated voxels in the occipital cortex was significantly smaller in SC subjects compared to EB subjects, both in the olfactory and in the auditory-verbal conditions.
(TIF)

Table S1 Table S1: Results from the behavioral experiment in blind and sighted subjects.
(DOC)

Table S2 Table S2 related to Figure 3: List of brain activation foci (positive values) obtained in the group comparison (EB>SC) during the olfactory and auditory-verbal conditions.
(DOC)

Table S3 Table S3 related to Figure 3: List of brain activation foci (positive values) obtained in the contrast between the olfactory and the auditory-verbal modality in EB subjects.
(DOC)

Table S4 Table S4 related to Figure 3: List of brain activation foci (positive values) obtained in the contrast between the olfactory and the auditory-verbal modality in SC subjects.
(DOC)
