# Supplementary figures and images for: Right Occipital Cortex Activation Correlates with Superior Odor Processing Performance in the Early Blind
Source: PLoS One. 2013 Aug 14;8(8):e71907. doi: 10.1371/journal.pone.0071907 (PMC3743806; doi:10.1371/journal.pone.0071907)

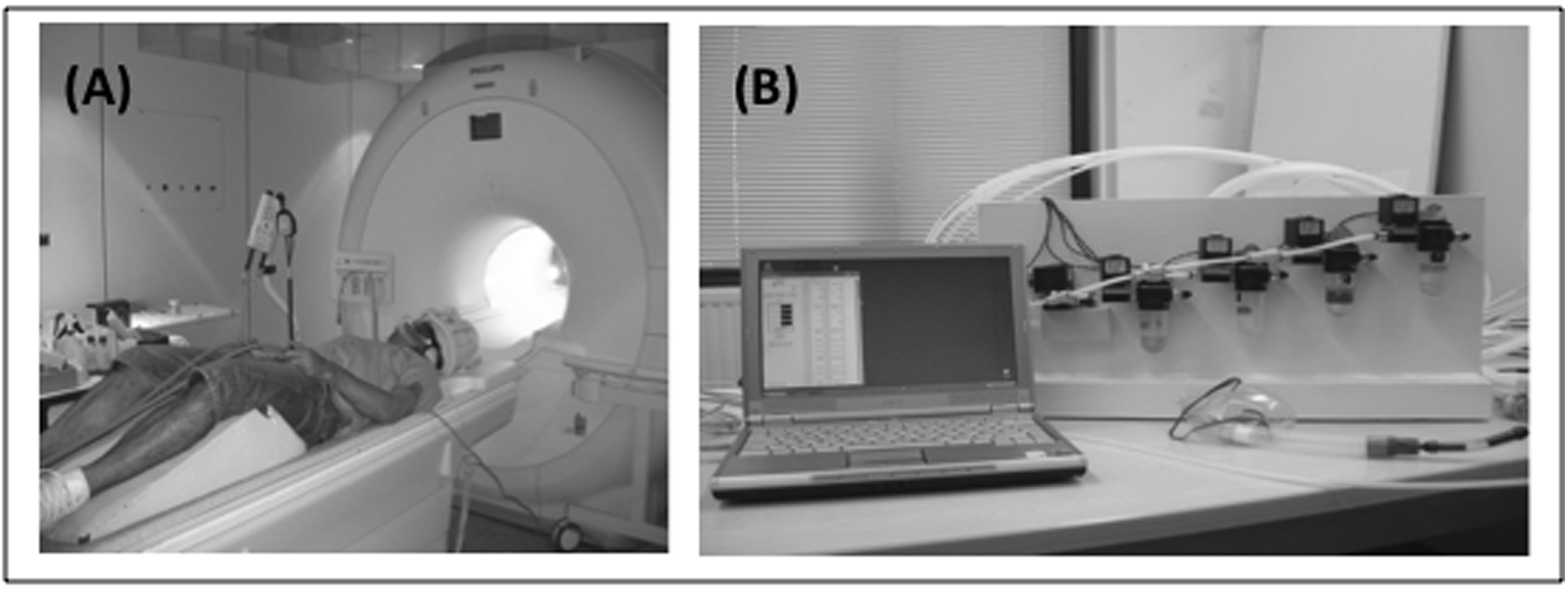

Supplement: Figure S1 — Olfactory stimulation equipment. (A) Image of a blindfolded sighted participant equipped with the odor delivery system in the fMRI room. Auditory signals were delivered via headphones to synchronize odor stimulations and breathing rhythm. (B) Detailed front view of the computer-controlled stimulator device showing the nylon channels, fittings and Teflon tube that deliver the switched air streams to the participant via a removable medical mask, as well as the solenoid valves and oil lubrificators containing the four different odorants in solution [42]. The main part of the device and the computer remained outside the fMRI room, whereas five Nylon channels passed to the fMRI room through a conventional security hole (see [42] for details). (TIF) [file pone.0071907.s001.tif]

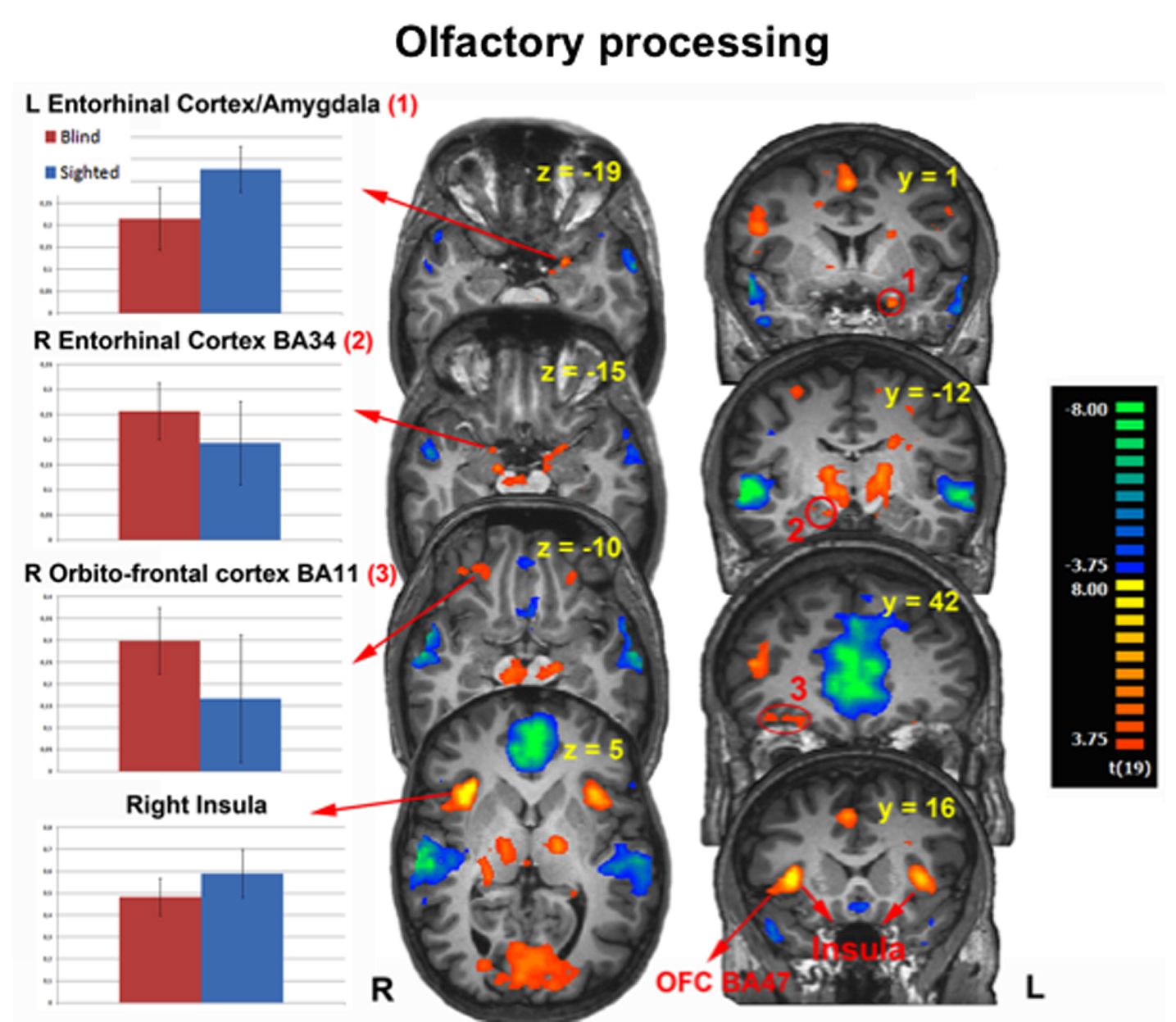

Supplement: Figure S2 — Brain activation observed during the olfactory processing. Activation maps resulting from olfactory conditions as compared to the baseline {Odor categorization+Odor discrimination versus Rest} in the whole group (both early blind (EB) and sighted control (SC) subjects grouped together: n = 20). To better circumscribe the activation foci in the olfactory regions shown in this Figure, we used a threshold of q<0.01 (FDR corrected). Brain activation foci were superimposed on the axial and coronal sections of an individual normalized MRI brain. Significant differences in this contrast (random-effects (RFX) analysis) are coded using a color scale of the t-values. Activation foci were found in the left and right entorhinal cortex/amygdala, which is considered as a part of the primary olfactory cortex [63], [77]–[78] . Additional activation foci were found in the secondary olfactory cortex: the orbito-frontal cortex (OFC, BA11) and the insula/OFC (BA13-47) bilaterally. In the left and right entorhinal cortex, the cluster size were 233 and 17 voxels. The activation foci in the OFC were located in three sub-regions: BA10 in the right hemisphere (x = 37, y = 53, z = 4; 220 voxels), BA11 bilaterally (x = 24, y = 41, z = −10; 333 voxels and x = −28, y = 38, z = −11; 189 voxels) and BA47 bilaterally. The activation foci in BA47 were included in larger clusters that covered most of the insula (x = 37, y = 17, z = 5; 6959 voxels and x = −37, y = 16, z = 7; 4315 voxels). The graphs on the left show the beta values for the olfactory conditions plotted together in four representative olfactory regions (the right entorhinal cortex: x = 17, y = −1, z = −15, 17 voxels, the left entorhinal cortex: x = −19, y = −3, z = −16, 233 voxels, the right OFC (BA11): x = 24, y = 41, z = −10, 248 voxels and the right insula: x = 37, y = 17, z = 5, 2577 voxels) as a function of the group. Error bars are standard errors of the means (s.e.m.). No group difference was observed in any region in the olfact [file pone.0071907.s002.tif]

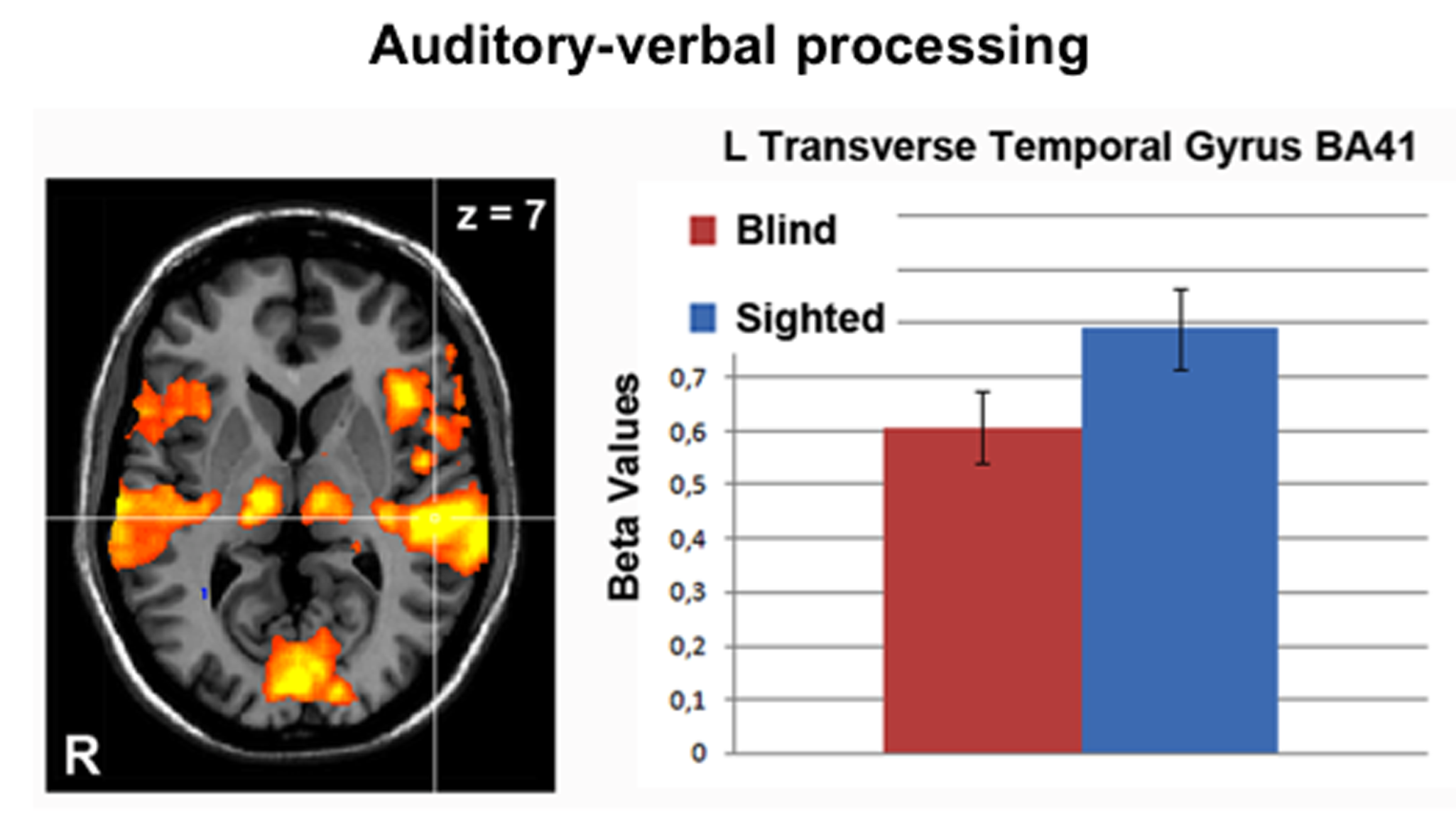

Supplement: Figure S3 — Brain activation observed during auditory-verbal processing. Activation maps resulting from auditory conditions as compared to the baseline {Word categorization+Word discrimination versus Rest} in the whole group (both EB and SC grouped together: n = 20) in random-effects analysis (RFX). To better circumscribe the activation foci in the auditory regions shown in this Figure, we used a threshold of q<0.05 corrected for multiple comparisons with a false discovery rate (FDR). Brain activation foci were superimposed on a transversal section of the normalized MRI brain of a representative subject. Activation foci are shown in the left and right primary and secondary auditory cortices (BA41, 42, 22) according to the color scale that codes the t-values. The lines intersect at coordinates (x = −50, y = −25, z = 7) on a voxel in the left transverse temporal gyrus (BA41). The graph on the right shows the beta values for the auditory conditions plotted together in a ROI of 604 voxels centered on the left transverse temporal gyrus (at the line intersection), as a function of the group. Error bars are standard errors of the mean (s.e.m.). No group difference was observed in any region in the auditory cortices though a trend was found in the left side, only (all p’s>0.05, see Results section). R: right. (TIF) [file pone.0071907.s003.tif]

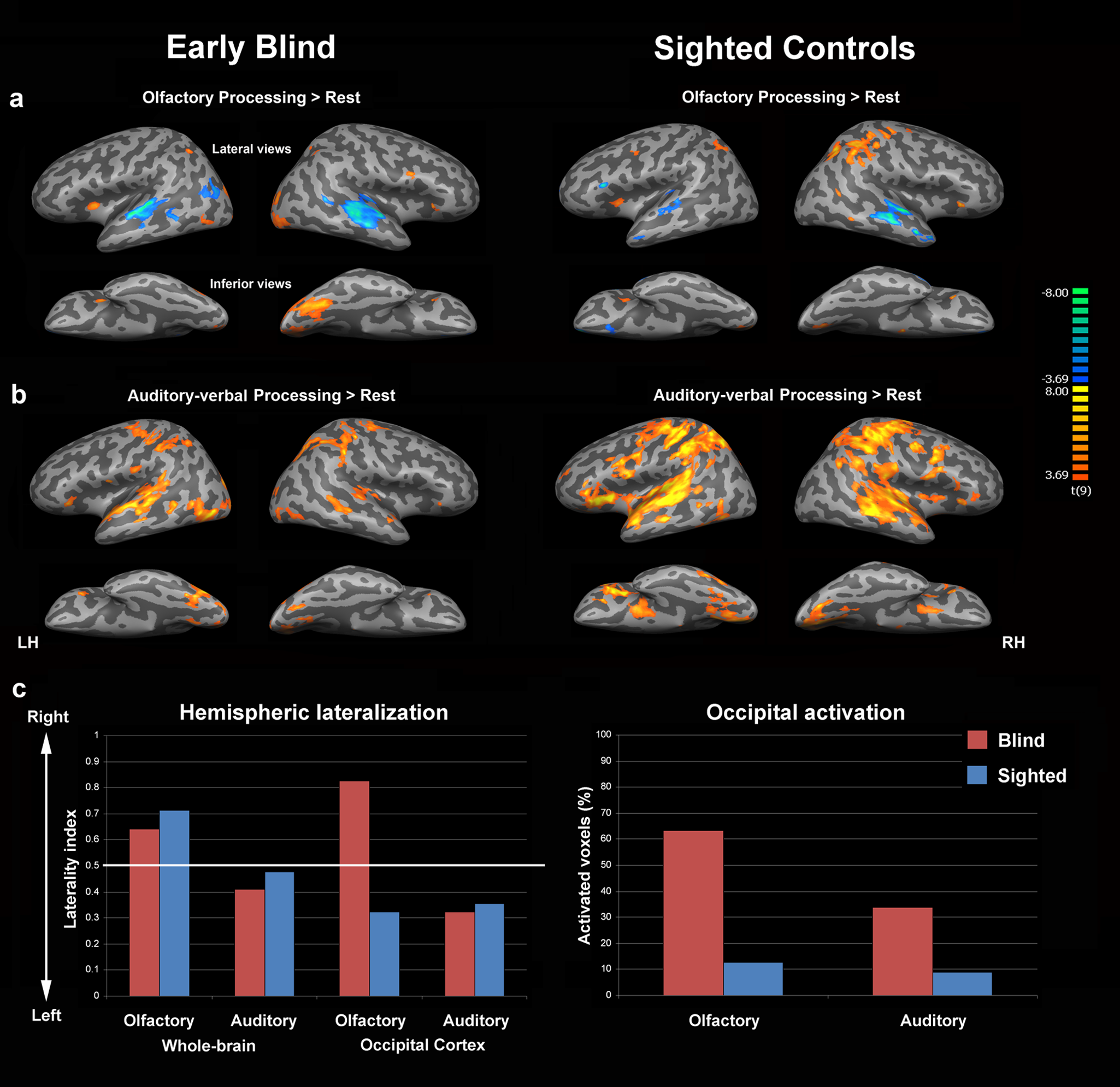

Supplement: Figure S4 — Brain areas recruited during olfactory and auditory-verbal processing in the group of blind subjects and the control group. Functional brain activity maps in 10 early blind subjects (EB) and 10 sighted control subjects (SC) during olfactory and auditory-verbal processing were projected onto a 3-D representation (inflated brain) of the right and left hemispheres (RH & LH) of a representative brain of one subject. The activation maps resulting from the contrasts between the olfactory and auditory-verbal conditions compared to the baseline (rest) were obtained using random-effects analyses (RFX) with a threshold of q<0.05, corrected for multiple comparisons using false discovery rate (FDR). (a) Brain activation related to olfactory processing in EB (left) and in SC subjects (right). (b) Brain activation related to auditory-verbal processing in EB (left) and in SC subjects (right). During the olfactory and auditory-verbal conditions, the occipital cortex was significantly activated in EB subjects and to a much lesser extent in SC subjects. Most activation foci observed in the OC of EB subjects were located mainly in the ventral stream in both sensory modalities. (c) The graphs show the laterality indexes (left) and the proportion of activated voxels within the occipital cortex relative to the number of activated voxels in the entire brain (right). Laterality indexes were similar to those used to measure the lateralization of functions such as language (e.g. [79]–[80]). The laterality indexes were calculated by dividing “x” by “x+y” (x/x+y), “x” corresponding to the number of voxels located in the right hemisphere as obtained in the contrasts (olfactory minus rest) and (auditory-verbal minus rest) and “y” corresponding to the number of voxels located in the left hemisphere as obtained in the same contrasts. These laterality indexes were calculated at ten different thresholds (between p = 0.05 and p = 0.000025) and averaged together. The mean laterality index was then plo [file pone.0071907.s004.tif]
