# Supplementary material for: Right Occipital Cortex Activation Correlates with Superior Odor Processing Performance in the Early Blind
Source: PLoS One. 2013 Aug 14;8(8):e71907. doi: 10.1371/journal.pone.0071907 (PMC3743806; doi:10.1371/journal.pone.0071907)
Supplement: Table S1 — Results from the behavioral experiment in blind and sighted subjects. (DOC) [file pone.0071907.s005.doc]

Table S1: Results from the behavioral experiment in blind and sighted subjects.

| ***Subjects*** | ***Age (years)*** | ***Odour discrimination***  ***(score /30)*** | ***Odour free-identification (score /30)*** | **Odour categorization** ***(score /30)*** | ***Mean performance (%)*** |
| --- | --- | --- | --- | --- | --- |
| EB1 | 23 | NA | 16 | 22 | NA |
| EB2 | 28 | 29 | 13 | 25 | 74 |
| EB3 | 31 | 28 | 11 | 20 | 66 |
| EB4 | 42 | 28 | 12 | 21 | 68 |
| EB5 | 57 | 30 | 15 | 21 | 73 |
| EB6 | 31 | 28 | 14 | 24 | 73 |
| EB7 | 43 | 29 | 10 | 20 | 66 |
| EB8 | 40 | 29 | 14 | 25 | 76 |
| EB9 | 52 | 28 | 14 | 23 | 72 |
| EB10 | 48 | 27 | 11 | 23 | 68 |
| SC1 | 22 | 26 | 8 | 20 | 60 |
| SC2 | 28 | NA | NA | NA | NA |
| SC3 | 31 | 28 | 7 | 21 | 62 |
| SC4 | 42 | 25 | 8 | 13 | 51 |
| SC5 | 55 | NA | NA | NA | NA |
| SC6 | 29 | 27 | 5 | 21 | 59 |
| SC7 | 42 | 21 | 5 | 18 | 49 |
| SC8 | 41 | 27 | 7 | 18 | 58 |
| SC9 | 51 | 24 | 7 | 22 | 59 |
| SC10 | 48 | 25 | 4 | 19 | 53 |

Note: EB: early blind; SC: sighted controls; all subjects were male and right handed; sighted subjects were studied blindfolded. Behavioral performance (number of correct answers) in a variety of higher-level odor processing tasks was averaged and expressed in percentage to provide a global score of odour-recognition ability before fMRI (see text). This global score (mean performance, %) was used in the covariance and correlation analyses. It should be noted that the correlation with each subscore was also significant (all p’s <0.01). NA: data incomplete or not available (subjects SC2 and SC5 were not tested; odour discrimination score of subject EB1 (30/30) was not recorded at the time of fMRI study).
