# Supplementary material for: Right Occipital Cortex Activation Correlates with Superior Odor Processing Performance in the Early Blind
Source: PLoS One. 2013 Aug 14;8(8):e71907. doi: 10.1371/journal.pone.0071907 (PMC3743806; doi:10.1371/journal.pone.0071907)
Supplement: Table S2 — related to Figure 3: List of brain activation foci (positive values) obtained in the group comparison (EB>SC) during the olfactory and auditory-verbal conditions. (DOC) [file pone.0071907.s006.doc]

Table S2 related to Figure 3 : List of brain activation foci (positive values) obtained in the group comparison (EB>SC) during the olfactory and auditory-verbal conditions.

| EB > SC (olfactory conditions) |  |  |  |  |  |  |  |  |  |  |  |  |
| --- | --- | --- | --- | --- | --- | --- | --- | --- | --- | --- | --- | --- |
| Brain region | BA | Mean x | Mean y | Mean z | t value | p value (peak) | | (x, y, z) (peak) | | | Cluster size | |
|  |  |  |  |  |  |  |  |  |  |  |  |  |
| R Insula | BA13 | 42,38 | 5,51 | -6,94 | 4,892821 | 0,000101 |  | 41 | 7 | -6 | 63 | * |
| R Claustrum |  | 35,49 | 4,98 | -0,77 | 4,752017 | 0,000139 |  | 35 | 4 | 0 | 43 | * |
| R Fusiform Gyrus BA19 | BA19 | 24,23 | -66,86 | -13,67 | 5,227783 | 0,000048 |  | 23 | -74 | -16 | 404 |  |
| R Inferior Frontal Gyrus | BA47 | 24,73 | 16,42 | -11,88 | 4,288137 | 0,000397 |  | 26 | 16 | -12 | 26 | * |
| L Anterior Cingulate | BA32 | -9,5 | 35,42 | 16,71 | 4,890186 | 0,000102 |  | -10 | 34 | 18 | 96 | * |
| L Inferior Frontal Gyrus | BA45 | -58,38 | 24,88 | 7,71 | 4,453531 | 0,000272 |  | -58 | 26 | 6 | 24 | * |
|  |  |  |  |  |  |  |  |  |  |  |  |  |
| EB > SC (auditory-verbal conditions) | |  |  |  |  |  |  |  |  |  |  |  |
| Brain region | BA | Mean x | Mean y | Mean z | t value | p value (peak) | | (x, y, z) (peak) | | | Cluster size | |
|  |  |  |  |  |  |  |  |  |  |  |  |  |
| L Middle Occipital Gyrus | BA19 | -46,45 | -72,91 | -5 | 4,066414 | 0,000658 |  | -52 | -69 | -3 | 22 | * |
|  |  |  |  |  |  |  |  |  |  |  |  |  |

R= right, L= left ; BA = Brodmann area ; p (uncorrected) < 0.001 with a cluster size threshold of p<0.05 ; * did not survive the cluster size threshold.
