# Supplementary material for: Right Occipital Cortex Activation Correlates with Superior Odor Processing Performance in the Early Blind
Source: PLoS One. 2013 Aug 14;8(8):e71907. doi: 10.1371/journal.pone.0071907 (PMC3743806; doi:10.1371/journal.pone.0071907)
Supplement: Table S3 — related to Figure 3: List of brain activation foci (positive values) obtained in the contrast between the olfactory and the auditory-verbal modality in EB subjects. (DOC) [file pone.0071907.s007.doc]

Table S3 related to Figure 3 : List of brain activation foci (positive values) obtained in the contrast between the olfactory and the auditory-verbal modality in EB subjects.

| EB : Olfactory minus Auditory |  |  |  |  |  |  |  |  |  |  |  |  |
| --- | --- | --- | --- | --- | --- | --- | --- | --- | --- | --- | --- | --- |
| Brain region | BA | Mean x | Mean y | Mean z | t value | p value (peak) | | (x, y, z) (peak) | | | Cluster size | |
|  |  |  |  |  |  |  |  |  |  |  |  |  |
| R Fusiform Gyrus | BA19 | 29,23 | -64 | -13,14 | 6,788552 | 0,00008 |  | 29 | -65 | -12 | 65 | * |
| R Mammillary Body |  | 5,62 | -9,86 | -10,31 | 9,556692 | 0,000005 |  | 5 | -11 | -9 | 105 | * |
| L Caudate Nucleus |  | -23,93 | -9,7 | 25 | 6,072919 | 0,000185 |  | -22 | -8 | 24 | 57 | * |
|  |  |  |  |  |  |  |  |  |  |  |  |  |
|  |  |  |  |  |  |  |  |  |  |  |  |  |
| EB : Auditory minus Olfactory |  |  |  |  |  |  |  |  |  |  |  |  |
| Brain region | BA | Mean x | Mean y | Mean z | t value | p value (peak) | | (x, y, z) (peak) | | | Cluster size | |
|  |  |  |  |  |  |  |  |  |  |  |  |  |
| R Superior temporal gyrus | BA22 | 57,39 | -25,16 | 3,29 | 12,523184 | 0,000001 |  | 59 | -32 | 3 | 8411 |  |
| R Inferior frontal gyrus | BA47 | 55,74 | 3,82 | -5,84 | 6,615788 | 0,000097 |  | 53 | 7 | -6 | 373 |  |
| R Inferior frontal gyrus | BA45 | 50,86 | 20,88 | 5,14 | 7,859985 | 0,000025 |  | 50 | 19 | 6 | 207 |  |
| R Precentral Gyrus | BA4 | 38,39 | -21,87 | 57,9 | 7,567958 | 0,000034 |  | 44 | -29 | 57 | 931 |  |
| R Postcentral gyrus | BA3 | 23,07 | -26,55 | 61,22 | 7,116431 | 0,000056 |  | 23 | -26 | 63 | 69 | * |
| R-L Medial Frontal Gyrus | BA10 | 1,63 | 56,23 | 10,29 | 6,079402 | 0,000184 |  | -4 | 58 | 12 | 348 |  |
| R-L Paracentral Lobule | BA5 | 3,19 | -14,9 | 45,42 | 5,456935 | 0,000402 |  | 2 | -14 | 45 | 31 | * |
| L Superior Frontal Gyrus | BA10 | -13,39 | 58,97 | 24,28 | 5,86779 | 0,000238 |  | -13 | 58 | 24 | 36 | * |
| L Postcentral Gyrus | BA3 | -41,38 | -23,4 | 52,9 | 7,058136 | 0,000059 |  | -43 | -23 | 54 | 223 |  |
| L Superior/Middle Temporal Gyrus | BA22 | -58,76 | -26,04 | 2,88 | 20,151285 | 0 |  | -58 | -26 | 6 | 11432 |  |
| L Superior Temporal Gyrus | BA22 | -54,65 | 12,75 | -4,51 | 7,011224 | 0,000062 |  | -55 | 10 | -6 | 477 |  |
| L Inferior Temporal Gyrus/Middle Occipital Gyrus | BA37/19 | -54,98 | -61,29 | -1,81 | 5,538164 | 0,000362 |  | -55 | -59 | -3 | 130 |  |
|  |  |  |  |  |  |  |  |  |  |  |  |  |

R= right, L= left ; BA = Brodmann area ; p (uncorrected) < 0.001 with a cluster size threshold of p<0.05 ; * did not survive the cluster size threshold.
