# Supplementary material for: Right Occipital Cortex Activation Correlates with Superior Odor Processing Performance in the Early Blind
Source: PLoS One. 2013 Aug 14;8(8):e71907. doi: 10.1371/journal.pone.0071907 (PMC3743806; doi:10.1371/journal.pone.0071907)
Supplement: Table S4 — related to Figure 3: List of brain activation foci (positive values) obtained in the contrast between the olfactory and the auditory-verbal modality in SC subjects. (DOC) [file pone.0071907.s008.doc]

Table S4 related to Figure 3 : List of brain activation foci (positive values) obtained in the contrast between the olfactory and the auditory-verbal modality in SC subjects.

| SC : Olfactory minus Auditory |  |  |  |  |  |  |  |  |  |  |  |  |
| --- | --- | --- | --- | --- | --- | --- | --- | --- | --- | --- | --- | --- |
| Brain region | BA | Mean x | Mean y | Mean z | t value | p value (peak) | | (x, y, z) (peak) | | | Cluster size | |
|  |  |  |  |  |  |  |  |  |  |  |  |  |
| R Pulvinar |  | 24,86 | -27,09 | 8,35 | 6,333134 | 0,000136 |  | 26 | -26 | 9 | 43 | * |
| R Cerebellum |  | 3,32 | -51,61 | -19,74 | 7,283161 | 0,000046 |  | 2 | -53 | -18 | 309 |  |
| L Cerebellum |  | -9,53 | -50,05 | -25,93 | 5,603207 | 0,000333 |  | -13 | -50 | -27 | 43 | * |
| L Medial Frontal Gyrus | BA6 | -15,94 | 3,62 | 46,88 | 6,243812 | 0,000151 |  | -16 | 4 | 48 | 32 | * |
|  |  |  |  |  |  |  |  |  |  |  |  |  |
| SC : Auditory minus Olfactory |  |  |  |  |  |  |  |  |  |  |  |  |
| Brain region | BA | Mean x | Mean y | Mean z | t value | p value (peak) | | (x, y, z) (peak) | | | Cluster size | |
|  |  |  |  |  |  |  |  |  |  |  |  |  |
| R Superior Temporal Gyrus | BA21-22 | 54,28 | -14,32 | -0,11 | 18,360603 | 0 |  | 59 | -8 | -3 | 12952 |  |
| R Inferior Frontal Gyrus | BA47 | 49,87 | 20,76 | 0,22 | 7,800423 | 0,000027 |  | 47 | 19 | -3 | 274 |  |
| R Postcentral Gyrus | BA3 | 41,82 | -28,86 | 47,45 | 6,415999 | 0,000123 |  | 44 | -29 | 51 | 65 | * |
| R Precentral Gyrus | BA4 | 32,85 | -24,22 | 57,37 | 6,269869 | 0,000146 |  | 32 | -23 | 57 | 122 |  |
| R Putamen |  | 29,05 | -9,42 | 2,41 | 6,560494 | 0,000104 |  | 29 | -11 | 3 | 81 | * |
| L Medial Frontal Gyrus | BA9 | -2,17 | 43,86 | 16,94 | 17,518974 | 0 |  | 2 | 46 | 18 | 13197 |  |
| R-L Paracentral Lobule | BA5 | -1,27 | -19,56 | 47,28 | 10,245461 | 0,000003 |  | -1 | -35 | 57 | 4337 |  |
| R Cingulate Gyrus | BA31 | 1 | -36,18 | 31,57 | 6,79757 | 0,000079 |  | 2 | -38 | 30 | 68 | * |
| L Cingulate Gyrus | BA31 | -8,93 | -56,32 | 28,4 | 6,100678 | 0,000179 |  | -10 | -59 | 30 | 92 | * |
| L Precuneus | BA7 | -10,45 | -72,48 | 23,04 | 6,445905 | 0,000119 |  | -7 | -74 | 24 | 184 |  |
| L Caudate Body |  | -10,02 | 4,21 | 10,23 | 7,497324 | 0,000037 |  | -10 | 4 | 9 | 190 |  |
| L Middle Frontal Gyrus | BA8 | -24,4 | 24,62 | 39,57 | 7,131922 | 0,000055 |  | -25 | 22 | 39 | 68 | * |
| L Superior Temporal Gyrus | BA22 | -55,28 | -18,94 | 2,82 | 16,529257 | 0 |  | -43 | -26 | 12 | 14274 |  |
| L Postcentral Gyrus | BA2 | -47,96 | -25,35 | 48,47 | 8,298704 | 0,000016 |  | -49 | -26 | 42 | 1324 |  |
| L Inferior Frontal Gyrus | BA47 | -46,86 | 18,85 | -7,77 | 7,328975 | 0,000044 |  | -46 | 19 | -9 | 254 |  |
| L Inferior Frontal Gyrus | BA45 | -56,26 | 22,95 | 8,11 | 9,943783 | 0,000004 |  | -58 | 22 | 9 | 1085 |  |
| L Middle Temporal Gyrus | BA21 | -54,08 | 8,69 | -20,27 | 5,662293 | 0,000309 |  | -55 | 10 | -21 | 51 | * |
| L Precentral Gyrus | BA4 | -59,91 | -9,51 | 25,84 | 6,285082 | 0,000144 |  | -58 | -8 | 27 | 70 | * |

R= right, L= left ; BA = Brodmann area ; p (uncorrected) < 0.001 with a cluster size threshold of p<0.05 ; * did not survive the cluster size threshold.
